# ‘The Telepsychiatry Clinic On-the-Run’: How a Unique Tele-Mental Health Clinic Is Thriving in the Midst of Political Turmoil in Myanmar

**DOI:** 10.1192/bjo.2025.10528

**Published:** 2025-06-20

**Authors:** Nilar Thwin, Khin May Lwin, Paing Soe, Yan Lin Aung, Ei Thin Zar Thwe

**Affiliations:** 1 Ministry of Health, National Unity Government, Myanmar; 2 Maimonides Medical Center, Brooklyn, New York, USA; 3 Lancashire and South Cumbria NHS Foundation Trust, Blackburn, United Kingdom

## Abstract

**Aims:** The WHO estimated that 1 in 5 people living in conflict areas have some form of mental disorder. On top of this, limited access to mental health care in these areas continues to amplify the issue. However, hope can be found through the increased use of telehealth during the COVID-19 pandemic, which has opened the opportunity to apply the platform in conflict zones.

The pre-existing need of mental health services in Myanmar was aggravated by the 2021 coup and the ongoing COVID 19 pandemic. The Ministry of Health (MOH) of the National Unity Government (a chosen government formed with democratically elected MPs) developed a system of free telehealth care. The Tele Health Clinic was initially composed of healthcare workers from the liberated borders of Myanmar and later joined by local/international clinicians. The Tele Mental Health (MH) Clinic was then opened in August 2021 to address the devastating mental health burden superimposed by the combination of coup and pandemic.

Objective of the Study: To describe the development of a telepsychiatry clinic amidst the current political turmoil in Myanmar.

**Methods:** Review of the historical data of Myanmar civil war, military coup in 2021 and Civil Disobedience Movement.

Review of UNHCR data on the impact of current conflict.

Literature review of mental health in conflict zones and role of telepsychiatry in conflict settings.

Review of the process of development of the Tele Mental Health Clinic in Myanmar.

Descriptive analysis of one year data of the clinic.

**Results:** The Tele-Mental Health clinic was developed following the collapse of the healthcare system after the 2021 military coup in Myanmar. The clinic uses innovative ways to recruit clinicians and to provide psychiatric services. While there is ongoing risk of military junta’s persecution, arrest and torture, the clinic continues to thrive and expand.

**Conclusion:** Telepsychiatry can be used as a safe and effective way to bridge the barriers to mental health care throughout the current political turmoil in Myanmar. The evidence on the effectiveness, cost-effectiveness, process of implementation and long-term measures to overcome challenges are areas of future research interests.

